# Antiatherogenic Potential of *Nigella sativa* Seeds and Oil in Diet-Induced Hypercholesterolemia in Rabbits

**DOI:** 10.1093/ecam/neq071

**Published:** 2011-06-16

**Authors:** Ghanya Al-Naqeep, Adel S. Al-Zubairi, Maznah Ismail, Zulkhairi Hj Amom, Norhaizan Mohd Esa

**Affiliations:** ^1^Department of Nutrition and Dietetics, Faculty of Medicine and Health Sciences, Universiti Putra Malaysia, 43400, Serdang, Selangor, Malaysia; ^2^Laboratory of Molecular Biomedicine, Institute of Bioscience, Universiti Putra Malaysia, 43400, Serdang, Selangor, Malaysia; ^3^Department of Food Science & Technology, Faculty of Agriculture, University of Sana'a, Sana'a, Yemen; ^4^Laboratory of Cancer Research MAKNA-UPM, Institute of Bioscience, Universiti Putra Malaysia, 43400, Serdang, Selangor, Malaysia; ^5^Department of Biochemistry and Molecular Biology, Faculty of Medicine and Health Sciences, University of Sana'a, Sana'a, Yemen

## Abstract

*Nigella sativa* or Black seed (*N. sativa* L.) is traditionally used for several ailments in many Middle Eastern countries. It is an annual herbaceous plant that belongs to the Ranuculacea family with many beneficial properties as antitumor, antidiabetic, antihypertensive, antioxidative and antibacterial. This work attempted to study the effect of *N. sativa* seeds powder and oil on atherosclerosis in diet-induced hypercholesterolemic (HC) rabbits in comparison with simvastatin (ST). Twenty-five adult New Zealand male white rabbits, weighing 1.5–2.5 kg, were divided into five groups; normal group (NC, *n* = 5) and four hypercholesterolemic groups (*n* = 20): a positive control (PC) and three HC groups force fed diet supplemented with 1000 mg Kg^−1^ body weight of *N. sativa* powder (NSP), 500 mg Kg^−1^ body *N. sativa* oil (NSO) and 10 mg Kg^−1^ ST for 8 weeks. Feeding HC rabbits with *N. sativa* either in powder or oil forms was shown to significantly reduce (*P* < .05) total cholesterol (TC) and low-density lipoprotein cholesterol (LDLC) levels and enhance high-density lipoprotein cholesterol (HDL) levels after treatment for 2, 4, 6 and 8 weeks compared to the PC group. Plaque formation was significantly inhibited while the intima: media ratio was significantly reduced in the NSP and NSO supplemented groups compared to the PC group. In conclusion, treatment of HC rabbits with *N. sativa* seeds powder or oil showed hypocholesterolemic and antiatherogenic cardioprotective properties.

## 1. Introduction

Complementary and Alternative Medicine (CAM), including herbal medicine, is popular in the general population worldwide [1]. *Nigella sativa* L., commonly known as black seeds have been used for nutritional and medicinal purposes in many Middle Eastern countries and other parts of the world [2, 3]. Seeds oil of both *N. sativa* and Neem has been used traditionally in Asia and the Middle East to treat many diseases and several viral diseases [4, 5]. Recently, researchers have taken interest into the seeds in different forms: the seed itself, the seed extract, its oil and its volatile substances. Studies on *N. sativa* seed and its oil have provided scientific support for the traditional use of the seed and its oil for treatment of rheumatism, immune stimulation, diabetes, cancer and related inflammatory diseases [6]. *Nigella sativa* seeds active constituents, for example volatile oil and thymoquinone, showed protection against nephrotoxicity and hepatotoxicity induced by either diseases or chemicals [7]. The seed oil has anti-inflammatory, analgesic, antipyretic, antimicrobial and antineoplastic activity [7]. Evidence concerning the hypocholesterolemic effect of *N. sativa* seeds in animals and human is inconclusive.

Heart diseases remain one of the leading causes of death worldwide [8]. Several publications produced from different laboratories included recommendations for a reduction in cholesterol consumption as means of preventing these diseases [8]. These recommendations have gained support as the role of hypercholesterolemia in the incidence of coronary heart diseases has been established. In addition, most of the studies in this area shifted the attention to the ways of lowering plasma cholesterol. However, few studies have been carried out *in vivo* to investigate the hypocholesterolemic properties of *N. sativa* seeds and their oil. *Nigella sativa* seeds oil and thymoquinone have been shown to have a hypocholesterolemic activity in rats [6, 9]. There is no available study, which examines the potential of *N. sativa* in powder form and as a part of the diet on hypercholesterolemia. Furthermore, to our knowledge, no published study has investigated the antiatherogenic benefits of *N. sativa* seeds and oil. Therefore the aim of this study was, to investigate the hypocholesterolemic and antiatherogenic properties of *N. sativa* seeds and the extracted oil on diet-induced hypercholesterolemic in rabbits.

## 2. Methods

### 2.1. *Nigella sativa* Seeds Collection and Extraction

*Nigella sativa* seeds were purchased from a local herbal grocery from Taiz—a city— in the Republic of Yemen. The seeds were cleaned and kept at 4°C in the Laboratory of Molecular Biomedicine, Institute of Bioscience, University Putra Malaysia, Malaysia. The seeds were ground using an electric grinder (National, model MX-915 C Japan). Homogenized and ground samples (100 g) were soaked overnight with an *n*-hexane (Fisher Scientific Co Ltd, Ottawa, Canada) at a ratio of 1 :  5 (w/v) and filtered using the Whatman paper (Fisher Scientific Co Ltd). The solvent was evaporated in a vacuum rotary evaporator (Buchi, Flawil, Switzerland) at 40°C. The crude oil samples were combined, weighed, and stored at −30°C until analysis.

### 2.2. Experimental Animals and Diet

Twenty-five male New Zealand white rabbits weighing 1.25–2.5 kg were used for this study. They were housed individually in stainless steel mesh-bottomed cages and were fed initially standard rabbits chow pellets for 1 week for adaptation. All rabbits were maintained at almost constant environmental conditions throughout the study at 21–24°C and 12 h 1ight : dark cycle. This study was carried out according to the guidelines approved by the Animal Care and Use Committee (ACUC) of Faculty of Medicine and Health Sciences, Universiti Putra Malaysia.

### 2.3. Diet Ingredients and Preparation

The diet used was obtained from Miba Mannsura (Malaysia), containing: soybean meal (15%), corn (30%), palm kernel meal (36%), soybean oil (2%), starch (10%), molasses (2%), mineral mixture (3.5%), vitamins mixture (0.3%), DL-methionine (0.2%), calcium carbonate (CaCO_3_), (0.5%) and calcium phosphate (CaHPO_4_) (0.5%) (Table 1). Diet was mixed and prepared in the laboratory of nutrition in Animal Sciences Department, University Putra Malaysia. The ingredients (soybean, corn and PKC) were ground using an electric grinder (Manesty 3001 UK), weighed and mixed using an electric mixer (SPAR, 107). The mineral and vitamin mixtures, starch (10%) and oil and molasses were mixed with cholesterol (1%) and dried in an oven at 45–50°C overnight. 

### 2.4. Chemicals and Reagents

Cholesterol and Sudan 1V were purchased from Sigma-Aldrich, St Louis, Missouri, USA and Simvastatin was purchased from Ranbaxy, Pharmaniga Logisttics Sdn Bhd 260790-T, while TC, LDL, HDL and triglycerides (TG) estimation kits were supplied by Roche Diagnostic GmbH, d-68298 Mannheeim, Germany. Formalin was obtained from BHD Chemicals and Xylene from Ajax Chemicals, Auburn, Australia, whereas Hematoxylin and Eosin (H&E) were purchased from Merck, Amsterdam, The Netherlands. Absolute alcohol was bought from R&M Chemicals, Essex, UK.

### 2.5. Experimental Design

The rabbits were randomly assigned to five groups of five animals each; negative control (NC), received a normal diet prepared and used as reference and four groups induced with hypercholesterolemia (*n* = 20) in which rabbits were fed normal diet supplemented with 1% cholesterol for 3 weeks. Hypercholesterolemic rabbits were further subdivided into four groups: a positive control (PC) nontreated group, a cholesterol diet supplemented with 1000 mg kg^−1^ *N. satvia* seeds in powder form (NSP), a cholesterol diet supplemented with 500 mg kg^−1^ *N. sativa* seeds oil (NSO) and a group force-fed cholesterol diet supplemented with 10 mg kg^−1^ day^−1^ simvastatin (ST) (dissolved in 4 mL distilled water and was given orally) for 8 weeks. The amount of food and water was recorded daily, while the body weight was recorded every 2 weeks.

### 2.6. Lipid Profiles Analysis

Blood samples from the ear marginal vein of the rabbits were taken before and after 1.0% cholesterol administration for 3 weeks, and after 2, 4, 6 and 8 weeks of treatment. Analysis of lipid profiles was carried out using Hitachi Analyzer.

### 2.7. Aortic Inner Surface Lesion Formation

At the end of the experiment, rabbits were dissected and aortas were removed, opened longitudinally and were prepared for accurate detection and estimation of lipid deposits in the intima following the method reported by Prasad [10]. Photographs of the inner surface of the aorta were analyzed for the plaque area using image-analysis software. The aortic strips were immersed in 10% formalin for 24  h and rinsed briefly in 70% ethanol. The tissues were then immersed in Herxheimer's solution containing Sudan IV (5  g), ethyl alcohol 70% (500 mL) and acetone (500 mL) at room temperature for 15 min and were washed under running water for 1 h. The aortas were placed on plastic templates and the luminal surfaces were photographed using a digital camera. The total and atherosclerotic areas of the intimal surface of the aorta were measured in square millimeters using an image-analysis software. The aortic plaques were assessed blindly, in which the scorer does not know the samples to which group it belong, samples previously coded. The extent of atherosclerosis was expressed as a percentage of the luminal surface covered by atherosclerotic changes [11].

Histological analysis of the nearest part of aorta to the heart was cut, labeled and fixed in 10% formalin for a few days and prepared for light microscopy by dehydrating the tissue samples in an ascending series of alcohol solution for 14  h in an automatic tissue processor machine (TP1020). The tissues were blocked, and cut using a microtome (Letiz Wetzlar 1512) to 4-*μ*m sections, pasted on slides and dried on a hot plate at 50–55°C for 30 min and then kept at 37°C. H&E were used to stain the tissue sections. Plaque accumulation was analyzed for the average determination of the thickness of the intima, media and intima : media ratio for three rabbits per group using an image-analysis system.

### 2.8. Statistical Analysis

Data were presented as group means ± SD and were analyzed using SPSS program version 11.0. The differences between NC and hypercholesterolemic groups were tested by independent sample *t*-test. One-way ANOVA followed by Dunnet's *Post hoc* test was used to compare the means of the PC and the treatment groups (*N. sativa* in powder form group, *N. sativa* oil group and ST group).

## 3. Results

### 3.1. Body Weight

Feeding rabbits with 1% cholesterol-supplemented diet for 3 weeks resulted in a significant increase (*P* < .05) in body weight compared to NC. Body weight of the PC and the NC groups were shown to be increased until the end of the experiment. On the other hand, after 2 weeks of treatment the body weight of NSO and NSP groups were shown to be slightly decreased and this phenomenon was continued until the end of experiment. Similar effect on body weight was observed when animals treated with ST, but this effect started after 4 weeks of treatment.

### 3.2. Hypercholesterolemia Induction in Rabbits

**Figure 1:**
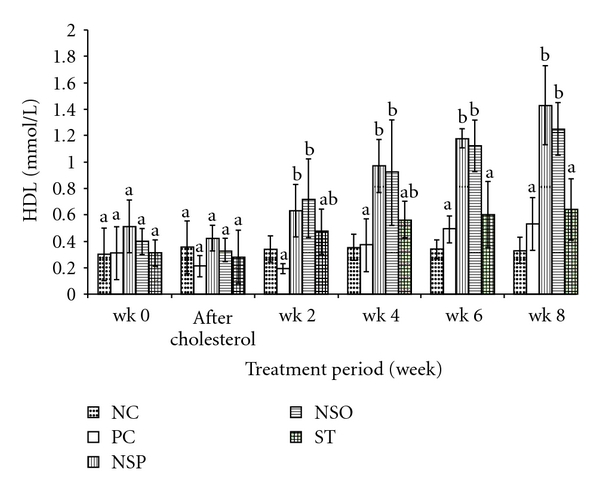
Changes in plasma HDL levels in 0 week, after induction 1% cholesterol for 3 weeks and after 2,4, 6 and 8 weeks of treat. Results are expressed as means ± SD of five animals per group. NSP, *Nigella satvia* seeds in powder form; wk, week. Values with the same superscript letters are not significantly different from each other at *P* < .05. Comparison of plasma HDL (mmol L^−1^) values at various times.

Feeding rabbits 1% cholesterol supplemented diet resulted in a significant increase in plasma TC and LDL levels and slightly decreased HDL levels (Tables 2 and 3 and Figure 1). In addition, these animals had plaque formation in the abdominal aorta, which led to a significant increase (*P* < .05) in the thickness of the intima and the intima : media ratio in cholesterol group as compared with the NC group. 

### 3.3. Plasma Lipid Profile Levels after *N. sativa* Seeds and Oil and ST Treatments in Experimental Rabbits

A significant reduction (*P* < .05) in plasma TC, LDL and TG levels of NSP-, NSO- and ST-treated groups were observed at 2, 4, 6 and 8 weeks of treatment compared to PC group (Tables 2–4) . Treatment of rabbits with NSP and NSO was shown to have a significant increase (*P* > .05) in plasma HDL levels as compared to the PC group at Weeks 4, 6 and 8 of treatment. However, throughout the treatment period no significant differences were noticed in plasma HDL and TG levels obtained from ST and PC groups. Plaque formation was significantly inhibited and the intima : media ratio was significantly reduced (*P* < .05) in *N. sativa* treatment groups and ST group as compared to the PC group. It should be mentioned that treatment with ST-induced mortality in rabbits (two rabbits) after 10 days of treatment and increased liver weight and its percentage to body weight. 

### 3.4. Assessment of Atherosclerotic Plaques

**Figure 2:**
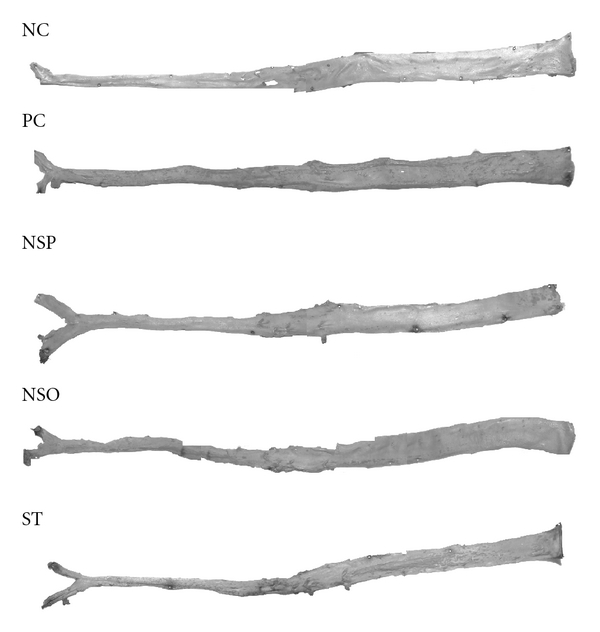
Representatives photographs of the intimal surfaces of the aortas from the five experimental groups showing Sudan IV-stained lipid deposits. Lipid deposits are stained brick-red.

Feeding rabbits 1% cholesterol supplemented diet-induced plaque formation in the abdominal aorta. The plaque area exceeded 10.63 ± 1.44%, 2.53 ±  1.06%, 2.19 ±  0.88% and 3.97 ± 0.47% in the PC, NSP, NSO and ST groups, respectively (Table 5). Lipid deposits in the intimal surface of the aorta of all groups were observed in which it was stained brick red when immersed into Sudan IV (Figure 2). Plaque formation in the whole area of the aorta was significantly inhibited (*P* < .05) by *N. sativa* and ST treatments as compared to the PC group. There was no significant difference (*P* > .05) in the whole area examined between treatment groups (NSP, NSO and ST). 

### 3.5. Quantitative Analysis of Histological Data

#### 3.5.1. Intima and Media Thickness

**Figure 3:**
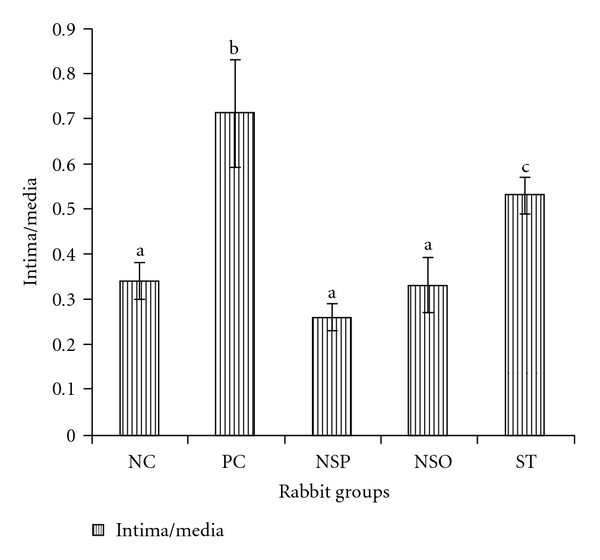
The intima : media ratio of the different groups compared to the NC group. NSP, *Nigella satvia* seeds in powder form. Values are means ± SD (*n* = 5). Values with the same superscript letters are not significantly different from each other (*P* < .05).

Thickness of the intima in the PC group was significantly higher (*P* < .05) when compared to NC group (Table 6 and Figure 3), but no significant differences were observed in the thickness of intima of the aorta in the treatment groups (NSP, NSO and ST) compared to NC. The PC group has shown to have the highest value of the intima thickness, but it was significantly different only when compared with the NSP and the NSO groups. On the other hand, significant reduction (*P* < .05) in the intima thickness of the NSP and the NSO groups in comparison with that result obtained from the ST-treated group. The new findings showed that there were no significant differences observed among all the treated groups compared to the NC group. 

#### 3.5.2. Intima : Media Ratio

**Figure 4:**
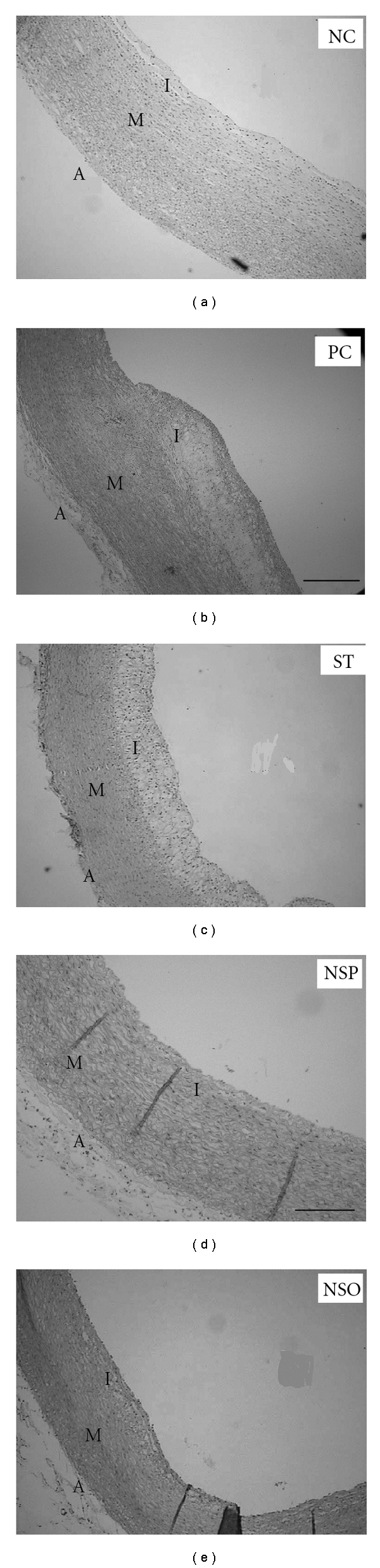
Representative photographs of the microscopic changes from the five groups stained with E&H stain.

The intima : media ratio in the animals fed cholesterol was significantly higher (*P* < .05) compared to the NC group, (Table 6 and Figure 4). The present results showed a significant difference (*P* < .05) between the PC group (71%) and the treatment groups (26, 33 and 53% in the NSP, NSO and ST groups, resp.). However, the intima : media ratio was observed to be significantly reduced in the NSP and NSO groups (*P* < .05) compared to the ST group, while there was no significant difference (*P* < .05) in intima : media ratio observed between the NSO and the NSP group. 

## 4. Discussion

Complementary and alternative medicine (CAM) has gained a worldwide popularity over the past 20 years [12, 13]. *Nigella sativa* seeds have been used for nutritional and medicinal purposes in many Middle Eastern countries and other parts of the world [1, 14, 15]. The seeds are considered a natural food additive and a condiment. However, they are typically consumed mixed with honey, and in baking products or pastries. It is evident that for most of the hypocholesterolemic drugs to be effective, they must be used for several weeks. This may expose the patients to several side effects, especially liver injury [16]. Thus, research has focused on the use of natural products of plant origin for the prevention of heart diseases.

Our results showed that body weight of the PC group was significantly increased until the end of the experiment compared to the NC, whereas the *N. sativa* treatment groups (NSO and NSP) showed slight decrease in the body weight during the 8 weeks of the treatment time. These results are similar to what was found by Zaoui et al. [9], who observed a significant decrease (*P* < .05) in body weight of normal rats that received a daily dose of 1 mg kg^−1^ of *N. sativa* fixed oil by oral gavage for 12 weeks. *Nigella sativa* treatment of rats (2 g kg^−1^ day^−1^ of the original seed for 1 week) reported to cause a reduction in the body weight accompanied by significant and sustained reduction in food intake [11]. ST group showed a decrease in body weight starting from Week 4 of the treatment until the end of the experiment, which might be resulted from the decreased daily food intake by 50% during the treatment time. These findings are similar to what was reported by Zagoya et al. [17], where they observed a decrease in food intake with weight loss of mice treated with statin.

Significant elevation in plasma TC and LDL levels and slight decrease in plasma HDL levels were used as indicators of hypercholesterolemia resulted from feeding rabbits cholesterol supplemented diet. These findings were in the same line as with those results reported by Prasad [10]. In addition, the significant reduction of TC and LDL levels and enhancement of HDL levels due to *N. sativa* treatments (NSO and NSP), are in agreement with the previous studies as reported by El-Dakhakhani et al. [2, 14], who found that feeding rats with *N. sativa* oil (800 g kg^−1^ day^−1^) orally for 4 weeks caused significant decreases in the serum LDL and TG levels, and an elevation of serum HDL levels. Recently, it was reported that the petroleum ether extract of *N. sativa* significantly reduced plasma TG and increased HDL cholesterol [11]. The volatile oil of *N. sativa* was observed to be as efficient as the cholesterol-reducing drug ST [18]. Furthermore, a study in hypercholesterolemic rats showed that feeding rats with *N. sativa* oil decreased serum TC, TG and LDL levels [9]. Additionally, treating rats with an oral dose of 1 mL kg^−1^ body weight of *N. sativa* seeds fixed oil for 12 weeks showed a significant decrease in total serum TC and TG [9].

On the other hand, our previous results [19] showed that *N. sativa* seeds oil is rich in vitamin E and total antioxidant activity, which may explain the significant reduction in plasma TC, LDL levels. As shown by Jorge et al. [20] vitamin E administered to hypercholesterolemic rabbits significantly reduced the plasma LDL and vessel wall oxidation after 2 and 4 days of treatment, respectively, which was associated with a decrease in vessel and plasma TC levels and an improvement in endothelial cell functioning after 6 days [20]. It was also found that oil extracted from *N. sativa* seeds is rich in unsaturated fatty acids, which could be responsible for the decrease of TC and LDL cholesterol levels as reported by other researchers [10]. The hypocholesterolimic effect of *N. sativa* seeds and their oil could be attributed to the seeds contents of total dietary fiber (TDF), insoluble dietary fiber (IDF) and soluble dietary fiber (SDF) as observed by Al-Nageeb et al. [21]. In addition, it was found that several dietary fibers significantly decrease plasma cholesterol levels in human subjects and thereby may reduce the risk of coronary heart diseases [22]. The present study demonstrated that ST treatment (10 g kg^−1^ day^−1^) significantly decreased TC and LDL levels when compared to the PC group over the treatment period and these findings are in agreement with those results obtained by other researchers [23, 24].

The results obtained from this study showed that feeding rabbits with 1.0% of cholesterol supplemented to their diet induced a significant increase in the lesions as compared to normal rabbits. In addition, our results showed that the plaque formation in all treatment groups was significantly inhibited as compared to the PC group. The reduction in atherogenesis caused by *N. sativa* seeds could be attributed to their high content of vitamin E, since increased consumption of vitamin E is inversely correlated with the development of the coronary heart diseases [16]. Furthermore, vitamin E supplementation significantly reduced atherosclerotic lesions in the ascending aorta of diet-induced hypercholesterolemic rabbits [25]. In addition to vitamin E, the antiatherogenic benefits of *N. sativa* treatment (NSP and NSO) may also be attributed to the active constituent of *N. sativa* seeds (Thymoquinone) as reported recently by Ragheb et al. [26] as well as to the high content of unsaturated fatty acids, where it has been shown that increased consumption of polyunsaturated fatty acids improves endothelium dependent relaxation and protects against the development of atherosclerotic cardiovascular diseases [10].

**Figure 5:**
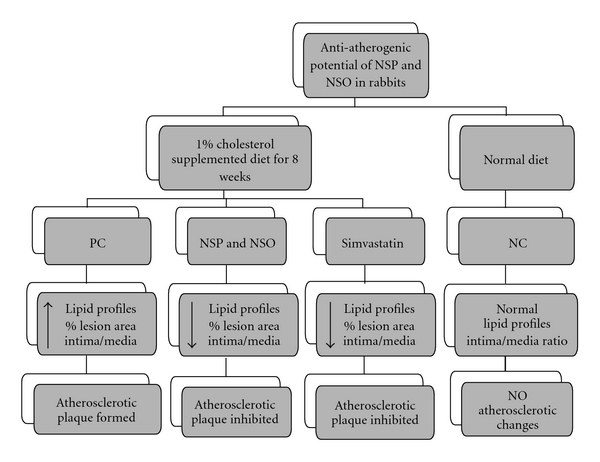
A hypothetical diagram to present the anti-atherogenic potential of *Nigella stiava* seeds and oil.

Feeding rabbits with 1% cholesterol supplemented diet-induced atherosclerosis; however, the histological examination of normal rabbit revealed that the aorta wall has a uniform thickness with no bulging in the lumen, and the intima was intact without any interruption in contrast to the hypercholesterolemic rabbits. Data from histopathological examination of *N. sativa* fed rabbits revealed a significant inhibition of aortic atherosclerotic changes when compared with the aorta of the PC group. In general *N. sativa* treatment (NSP and NSO) showed significant decrease in intima : media ratio compared to ST group. It was reported that, ST-reduced atherosclerotic plaque size and significantly increased the plaque content of vascular smooth muscle cells and collagen and reduced inflammation contributing to atherosclerotic plaque [27]. ST decreased the intima thickness and significantly decreased the intima : media ratio by 42 and 25%, respectively, compared to the PC group [27]. The relevance of these finding as shown in Figure 5 depict the beneficial role of *N. Sativa* powder and oil in preventing the development of atherosclerosis. 

In conclusion, this study points out to the importance of *N. sativa* seeds and oil in reducing the arterial wall lipid deposition, total cholesterol and LDL levels and consequently the atherogenesis indicating its potential health value.

## Funding

University Putra Malaysia (grant number 62166).

## Figures and Tables

**Table 1:** Diet ingredient prepared for the experimental rabbits.

Ingredient	Mixture of diet (%)
Soybean	15
Corn	30
Palm kernel meal	36
Starch	10
Molasses	2
Corn oil	2
Vitamins mixture^a^	0.3
Mineral mixture^b^	3.5
DL-methionine	0.2
CaCO_3_	0.5
CaHPO_4_	0.5

Total	100

^a^The vitamin premix provided (mg kg^−1^ feed): thiamin 60, riboflavin 22.5, niacinamide 152, calcium pantothenate 56, choline chloride 2000, inositol 1000, folic acid 8.5, biotin 1, pyridoxine-hydrochloride 22.5, *p*-aminobenzoic acid 500, Vitamin B12 0.015, DL-tocopheryl acetate 50, menadione 4, retinyl acetate and retinyl palmitate 30 (15  000 IU), cholecalciferol 30 (3000 IU), Vitamin C 400.

^b^The mineral premix provided (mg kg^−1^ feed): sodium citrate dihydrate, FeSO_4_ *·*7H_2_O 900, MnO_2_ 140, KAl(SO_4_)_2_·12H_2_O 200, ZnSO_4_·H_2_O 125, KBr 20, NiSO_4_·6H_2_O 8.5, CuSO_4_ *·*5H_2_O 100, CoSO_4_·7H_2_O 5, Na_2_MoO_4_·2H_2_O 5, KI 5, As_2_O_3_ 0.2, NaF 8.5, Na_2_B_4_O_7_·10H_2_O 5, Na_2_SeO_3_·5H_2_O.

**Table 2:** Changes in plasma TC levels (mmol L^−1^) at 0 week, after feeding of 1.0% cholesterol for 3 weeks and after 2, 4, 6 and 8 weeks of treatment.

Group	Week 0	HC induction for 3 weeks	Treatment period (weeks)			
			2	4	6	8
NC	0.81 ± 0.41^a^	1.0 ± 0.52^a^	1.29 ± 0.58	1.06 ± 0.40	0.88 ± 0.3	0.60 ± 0.26
PC	0.38 ±0.14^a^	10.33 ± 5.1^b^	15.09 ± 4.5^a^	13.09 ± 3.5^a^	8.23 ± 2.1^a^	5.41 ± 1.3^a^
NSO	0.48 ± 0.26^a^	10.04 ± 3.1^b^	7.15 ± 2.7^b^	3.59 ± 1.9^b^	1.62±0.8^b^	0.84 ± 0.3^b^
NSP	0.84 ± 0.8^a^	10.70± 5.4^b^	7.06 ± 3.8^c^	4.55 ± 2.0^b^	1.57 ± 0.7^b^	0.64 ± 0.3^b^
ST	0.31± 0.1^a^	12.90 ± 4.9^b^	9.90 ± 1.7 ^b,c^	6.72 ±1.7^b^	3.23 ± 1.5^b^	0.67 ± 0.6^b^

Results are expressed as means ±SD of five rabbits. All groups received 1% cholesterol added to the diet except for NC group. Within a column, values with the same superscript letters are not significantly different from each other at *P* < .05.

**Table 3:** Changes in plasma LDL levels (mmol L^−1^) at 0 week, after feeding of 1.0% cholesterol for 3 weeks and after 2, 4, 6 and 8 weeks of treatment.

Group	Week 0	HC induction for 3 weeks	Treatment period (weeks)			
			2	4	6	8
NC	0.36 ± 0.3 ^a^	0.56 ± 0.3^a^	0.36 ±0.2	0.37 ± 0.2	0.29 ± 0.1	0.24 ± 0.1
PC	0.22 ± 0.14^a^	9.20 ± 4.9^b^	12.79 ± 4.99^a^	10.92 ± 4.6^a^	6.49± 2.2^a^	3.88 ±1.6^a^
NSO	0.24 ± 0.2^a^	8.51 ±5.1^b^	4.46 ±2.2^b^	2.34 ±1.2^b^	1.06 ±0.6^b^	0.63 ±0.7^b^
NSP	0.43 ± 0.2^a^	9.31 ± 5.5^b^	5.28 ± 3.5^b^	2.33 ± 1.1^b^	1.16 ± 1.4^b^	0.28 ± 0.1^b^
ST	0.15 ±0.1^a^	12.14 ± 8.9^b^	6.76 ± 2.3^a,b^	4.95 ± 2.1^c^	2.18 ± 1.8^b^	0.42 ± 0.2^b^

Results are expressed as means ± SD of five rabbits. NC group was not given cholesterol. Within a column, values with the same superscript letters are not significantly different from each other at *P* < .05 Comparison of plasma LDL (mmol L^−1^) values at various periods.

**Table 4:** Changes in plasma TG levels (mmol L^−1^) at 0 week, after feeding of 1.0% cholesterol for 3 weeks and after 2, 4, 6 and 8 weeks of treatment.

Group	Week 0	HC induction for 3 weeks	Treatment period (weeks)			
			2	4	6	8

NC	0.57 ± 0.2^a^	0.64 ± 0.4^a^	0.54 ± 0.2	0.72 ± 0.2	0.52 ± 0.27	0.64 ± 0.4
PC	0.33 ± 0.07^b^	0.32 ±± 0.1^a^	0.61 ± 0.4^a^	1.02 ± 0.8^a^	0.79 ± 0.6^a^	0.42 ± 0.1^a^
NSO	0.22 ± 0.2^b^	0.37 ± 0.1^a^	0.60 ± 0.3^a^	0.30 ± 0.2^a^	0.54 ± 0.1^a^	0.40 ± 0.07^a^
NSP	0.34 ± 0.2^b^	0.72 ± 0.4^a^	0.51 ± 0.2^a^	0.46 ± 0.2^a^	0.50 ± 0.1^a^	0.40 ± 0.2^a^
ST	0.32 ± 0.2^b^	0.39 ± 0.1^a^	0.51 ± 0.2^a^	0.65 ± 0.4^a^	0.43 ± 0.2^a^	0.28 ± 0.1^a^

Within a column, values with the same superscript letters are not significantly different from each other at *P* < .05. Comparison of plasma TG (mmol L^−1^) values at various times.

**Table 5:** Percentage of lesion area in the experimental rabbits.

Group	Whole area of the aorta (cm^2^)	Area of the Atheroma (cm^2^)	%Lesion area
PC	7.53 ± 0.15	0.80 ± 0.12	10.63 ± 1.44^a^
NSP	6.62 ± 0.26	0.16 ± 0.07	2.53 ± 1.06^b^
NSO	7.21 ± 0.69	0.16 ± 0.47	2.19 ±0.88^b^
ST	6.71 ± 1.03	0.27 ±0.45	3.97 ± 0.47^b^

Effects of *N. sativa* and simvastatin treatments on plaque formation in the intraluminal surface of the abdominal aorta. Values are means ± SD (*n* = 5). Within a column, values with the same superscript letters are not significantly different from each other (*P* < .05).

**Table 6:** Maximum thickness of the intima, media and intima : media ratio in experimental rabbits.

Group	Intima thickness (*μ*m)	Media thickness (*μ*m)	Intima/media
NC	1257.02 ±437.68^a,c,b^	3791.9 ± 1357.65^a^	0.34 ± 0.04^a^
PC	3991.91 ± 1598.76^d^	5781.4 ± 2777.07^a^	0.71 ± 0.12^b^
NSP	1113.48± 487.22^b,a^	4208.82 ± 1393.89^a^	0.26 ± 0.03^a^
NSO	1660.40 ± 96.54^a^	4455.54 ± 679.94^a^	0.33 ± 0.06^a^
ST	2311.67 ± 730.72^c,d^	4298.86 ± 1157.51^a^	0.53 ± 0.04^c^

Values are expressed as means ± SD *n* = 5 for all groups. Within a column, values with the same superscript letters are not significantly different from each other (*P* < .05).
